# Circulating surfactant protein -D is low and correlates negatively with systemic inflammation in early, untreated rheumatoid arthritis

**DOI:** 10.1186/ar2948

**Published:** 2010-03-08

**Authors:** Anne Friesgaard Christensen, Grith Lykke Sørensen, Kim Hørslev-Petersen, Uffe Holmskov, Hanne Merete Lindegaard, Kirsten Junker, Merete Lund Hetland, Kristian Stengaard-Pedersen, Søren Jacobsen, Tine Lottenburger, Torkell Ellingsen, Lis Smedegaard Andersen, Ib Hansen, Henrik Skjødt, Jens Kristian Pedersen, Ulrik Birk Lauridsen, Anders Svendsen, Ulrik Tarp, Jan Pødenphant, Aage Vestergaard, Anne Grethe Jurik, Mikkel Østergaard, Peter Junker

**Affiliations:** 1 Department of Rheumatology, Odense University Hospital, Sdr. Boulevard 29, DK-5000 Odense C, Denmark and Institute of Clinical Research, University of Southern Denmark, Winsloewparken 19, DK-5000 Odense C, Denmark; 2 Medical Biotechnology Centre, University of Southern Denmark, Winsloewparken 25, DK-5000 Odense C, Denmark; 3 Department of Rheumatology, Rheumatism Hospital, Toldbodgade 3, DK-6300 Graasten, Denmark; 4 Department of Rheumatology, Copenhagen University Hospitals, Hvidovre and Glostrup, Kettegaards Alle 30, DK-2650 Hvidovre, Denmark; 5 Department of Rheumatology, Aarhus University Hospital, Noerrebrogade 44, DK-8000 Aarhus C, Denmark; 6 Department of Rheumatology, Copenhagen University Hospital, Rigshospitalet, Blegdamsvej 9, DK-2100 Copenhagen, Denmark; 7 Department of Rheumatology, Copenhagen University Hospitals, Herlev and Gentofte, Niels Andersens Vej 65, DK-2900 Hellerup, Denmark; 8 Department of Radiology, Copenhagen University Hospital, Hvidovre, Kettegaards Alle 30, DK-2650 Hvidovre, Denmark; 9 Department of Radiology, Aarhus University Hospital, Noerrebrogade 44, DK-8000 Aarhus C, Denmark

## Abstract

**Introduction:**

Surfactant protein D (SP-D) is a collectin with immuno-regulatory functions, which may depend on oligomerization. Anti-microbial and anti-inflammatory properties have been attributed to multimeric SP-D variants, while trimeric subunits per se have been suggested to enhance inflammation. Previously, we reported low circulating SP-D in early rheumatoid arthritis (RA), and the present investigation aims to extend these data by serial SP-D serum measurements, studies on synovial fluid, SP-D size distribution and genotyping in patients with early RA.

**Methods:**

One-hundred-and-sixty disease-modifying antirheumatic drug (DMARD) naïve RA patients with disease duration less than six months were studied prospectively for four years (CIMESTRA (Ciclosporine, Methotrexate, Steroid in RA) trial) including disease activity measures (C-reactive protein, joint counts and Health Assessment Questionnaire (HAQ) score), autoantibodies, x-ray findings and SP-D. SP-D was quantified by enzyme-linked immunosorbent assay (ELISA) and molecular size distribution was assessed by gel filtration chromatography. Further, SP-D Met11Thr single nucleotide polymorphism (SNP) analysis was performed.

**Results:**

Serum SP-D was significantly lower in RA patients at baseline compared with healthy controls (*P *< 0.001). SP-D increased slightly during follow-up (*P *< 0.001), but was still subnormal at four years after adjustment for confounders (*P *< 0.001). SP-D in synovial fluid was up to 2.5-fold lower than in serum. While multimeric variants were detected in serum, SP-D in synovial fluid comprised trimeric subunits only. There were no significant associations between genotype distribution and SP-D. Baseline SP-D was inversely associated to CRP and HAQ score. A similar relationship was observed regarding temporal changes in SP-D and CRP (zero to four years). SP-D was not associated to x-ray findings.

**Conclusions:**

This study confirms that circulating SP-D is persistently subnormal in early and untreated RA despite a favourable therapeutic response obtained during four years of follow-up. SP-D correlated negatively to disease activity measures, but was not correlated with x-ray progression or SP-D genotype. These observations suggest that SP-D is implicated in RA pathogenesis at the protein level. The exclusive presence of trimeric SP-D in affected joints may contribute to the maintenance of joint inflammation.

**Trial registration:**

(j.nr NCT00209859).

## Introduction

Within recent years, search for innate immune system abnormalities in rheumatoid arthritis (RA) has attracted considerable attention [1]. Thus, low serum levels of mannan-binding lectin (MBL) have been associated with increased risk of early disease onset and severity of RA [2,3]. Likewise, variant MBL alleles have been associated with an unfavourable disease course [4,5]. Recently, we reported that the serum level of another collectin, surfactant protein D (SP-D), is decreased in newly-diagnosed, untreated RA [6]. In that study comprising 45 DMARD naïve patients, systemic SP-D was not significantly associated to conventional measures of disease activity such as C-reactive protein and joint counts [6].

Collectins are pattern recognition molecules, which preferentially bind to carbohydrate moieties expressed on a variety of pathogens (pathogen associated molecular patterns (PAMPs)), thereby enhancing aggregation, opsonisation or MBL-mediated complement activation [7]. SP-D has a complex quaternary structure in which monomers are assembled into tetramers forming dodecamers or higher order multimers [8,9]. Multimeric SP-D is suggested to have anti-microbial properties [10-13]. The function of natural trimeric subunit SP-D is not known in detail, but it seems to be devoid of anti-inflammatory activity [10-13]. SP-D is primarily synthesized by the respiratory epithelium (type II epithelial cells and Clara cells) [14,15], but is also expressed in a variety of extra-pulmonary epithelia [16]. SP-D has been detected in various body fluids including serum, synovial fluid, lacrimal and broncho-alveolar lavage liquid [17-22]. A common polymorphism in the SP-D gene on chromosome 10, Met11Thr, resulting in either methionine or threonine at residue 11, is a major determinant for the serum concentration and multimerization of SP-D [13,22]. The Thr11-variant is associated with reduced oligomerization, reduced binding capacity of microbes and low serum levels in healthy subjects [13].

The present investigation extends our previous observation by readdressing the possible association between SP-D and the Met11Thr polymorphism in early, untreated RA, and by studying the correlation between SP-D and disease activity measures and radiographic progression during a four-year interventional study on DMARD naïve patients with RA of recent onset. In addition, we compared the SP-D molecular size distribution in synovial fluid and corresponding sera.

## Materials and methods

### Patients and controls

One-hundred-and-sixty RA patients were included in the multicenter, randomized, double-blinded, parallel-group, placebo-controlled CIMESTRA trial [23,24]. Briefly, patients fulfilled the American College of Rheumatology 1987 revised criteria for RA [25]. Further, the patients appeared with active disease less than six months, less than or equal to two swollen joints at baseline, and were aged 18 to 75 years [23,24]. Health Assessment Questionnaire (HAQ score, 0 to 3) [26], Visual Analogue Scale (0 to 10) (VAS pain, global and doctor) and Disease Activity Score in 28 joints (DAS28) [27] were calculated. Fourteen-hundred-and-seventy-six healthy twin-individuals aged 18 to 67 years served as controls [22]. The trial was approved by the local ethics committee (j. nr M1959-98) and fulfilled the Declaration of Helsinki and the International Conference on Harmonisation 1996 revised guidelines for Good Clinical Practice (j.nr NCT00209859). Signed informed consent was obtained from all study participants.

### Treatment strategy

The treatment protocol compared methotrexate (MTX) plus cyclosporine vs. MTX plus placebo. During the first eight weeks patients were assessed fortnightly and every four weeks thereafter. Subsequently, whenever synovitis was present MTX dose was escalated by 2.5 mg from 7.5 mg/week to maximum 20 mg/week followed by a stepwise cyclosporine/placebo-cyclosporine increment (0.5 mg/kg) every four weeks from 2.5 mg/kg to maximum 4.0 mg/kg. In addition, intra-articular betamethasone (7 mg/l) was injected into swollen joints at any visit (maximum four joints or 4 ml per visit). During the second year, hydroxychloroquine (200 mg/day) was added and cyclosporine/placebo was tapered to zero, while MTX was continued [23,24]. During the open extension study from three to four years the treatment strategy continued to aim at tight synovitis control. Oral glucocorticoids were allowed in the open extension study.

### Laboratory measures

Serum was obtained from routinely drawn non-fasting blood samples collected between 08.00 a.m. to 2.00 p.m. Samples were allowed to clot at room temperature followed by centrifugation at 3,000 × g for 10 minutes. Sera were stored at -80°C.

SP-D was measured at baseline, after two weeks, one and six months, and after one, two, three and four years using a five-layered sandwich ELISA as previously described [19]. In controls, SP-D was only measured at baseline. All analyses were done in duplicate and serial samples from the same patient were analyzed simultaneously. The inter-assay coefficients of variation were 3.5 and 3.8% for low (367 ng/ml) and high (2,470 ng/ml) quality controls, respectively, and the intra-assay coefficients of variation were 1.7% for both quality controls. C-reactive protein (CRP) (mg/l) and erythrocyte sedimentation rate (ESR) (mm/hour) were assayed by standard methods. IgM-rheumatoid factor (IgM-RF) (cut-off level < 16 IU/ml) and anti-CCP (cut-off level < 24 U/ml) (Euro Diagnostica AB, Malmö, Sweden) were measured by ELISA as previously described [28-30].

### Radiographic analysis

Radiographs of hands, wrists, and forefeet were obtained at baseline (n = 155), and annually thereafter. After four years 137 radiographs were available, but only 133 patients had radiographs available at baseline and at four years. Radiographs were scored according to Sharp-van der Heijde by an independent senior radiologist who was aware of the sequence of x-ray recordings [31]. The annual estimated progression rate in total Sharp-van der Heijde Score (TSS), Joint Space Narrowing score (JSN) and erosion score (ES) was calculated according to disease duration and TSS, JSN and ES at baseline for each patient [32]. Radiographic progression was defined as the smallest detectable difference from baseline (= one unit).

### Synovial fluid

Corresponding serum and synovial fluid samples were available from 20 RA patients with joint effusions before treatment. Synovial fluid was collected by aseptic technique before injection of glucocorticoid and stored at -80°C. Before analysis, the samples were centrifuged 30 minutes at 400 × g and subsequently the supernatant was incubated four hour at 37°C with bovine testicular hyaluronidase (Sigma H3884, St Louis, MO, USA) to reduce viscosity (2 μl hyaluronidase (1 mg/ml in 0.2 M TRIS, 0.1 M sodium acetate, pH 7.0) to 300 μl synovial fluid). Subsequently, they were centrifuged at 20.000 × g for 10 minutes at 4°C. The supernatant was assayed for SP-D by ELISA. The possible trapping of SP-D in the synovial fluid pellet was studied by incubating the pellet with ethylenediaminetetraacetic acid (EDTA) 0.52 M in a TRIS-buffered saline (TBS) buffer (pH 7.4) at 37°C in 30 minutes followed by centrifugation in four minutes at 20.000 × g and 4°C. A total of 50 μl of the resulting supernatant was re-calcified with 60 μl of 1 M CaCl_2_, and pH was adjusted to 7.9 by adding 28.5 μl 1 M TRIS pH 8.6 prior to analysis.

### Gel filtration chromatography

Gel filtration chromatography was done on available synovial fluid samples (n = 11) and corresponding sera. Hyaluronidase-treated samples (200 μl) were applied to an analytical Superose 6 column connected to a fast-performance liquid chromatography system (former Amersham Biosciences, now GE Healthcare, Uppsale, Sweden) using TBS (pH 7.4) containing 10 mM EDTA and 0.05% emulphogen as eluent at a flow rate of 24 ml/hr. Fractions of 0.2 ml were collected and quantified by the SP-D ELISA. SP-D was eluted as two structurally different forms with high and low molecular weight (SP-D multimers (fraction 10 to 18) and SP-D trimers (fraction 24 to 38)). Size chromatography on healthy serum followed by SDS-PAGE and Western blotting has yielded protein bands at > 250 kDa for multimeric SP-D, and 90 kDa, 43 kDa and 40 kDa for trimeric SP-D [13,19].

### Genotyping

Genomic DNA was isolated from EDTA stabilized whole blood. Applied Biosystems (Assay-by-design) (Foster City, California, USA) designed primers and probes for the non-synonymous substitutions of DNA-bases of the SP-D gene resulting in the Met/Thr variant. The genotyping procedure has been described previously [13]. Human leucocyte antigen (HLA)-DRB1 genotyping for shared epitope (SE) was performed by polymerase chain reaction-based sequence-specific oligonucleotide probing, as described elsewhere [33,34]. Herein, we define the shared epitope as the presence of HLA-DRB1*04 and/or HLADRB1*01 and/or HLADRB1*10.

### Statistical analysis

All statistical analyses were conducted using STATA version 9.2 (StataCorp, College Station, Texas, USA). Comparisons between groups were done by Mann-Whitney U-test or Fischer's Exact Test, and if analysing more than two groups, Kruskal-Wallis test was used. Spearman Rank Correlation analysis was applied when appropriate. Comparison between patients and controls was performed using linear regression models, where control twins were clustered in pairs. Linear regression was also applied in the prospective analysis of SP-D in RA patients, where repeated measurements in the individual patient were clustered. We used logistic regression to assess whether baseline SP-D could predict radiographic progression after four years with adjustment for gender, age, smoking, anti-CCP and radiographic status at baseline. Robust estimation of standard error was calculated. To approximate a normal distribution, SP-D was logarithmically transformed when used as continuous, dependent variable in linear regression analyses.

One individual from each healthy twin pair was used for genotype and allele frequency estimation. The genotype frequencies were tested for Hardy-Weinberg equilibrium by (χ^2^-analysis. Comparisons of genotype and allele frequencies in patients and controls were performed by logistic regression with adjustment for gender and age or by Fishers Exact test.

Since SP-D did not differ between treatment arms, data from all RA patients were pooled. Analysis was by intention-to treat (N = 142). Completers' analysis was also performed and gave similar results (data not shown). Results are presented as median (95% confidence interval) if not otherwise stated. *P*-values ≤ 0.05 and *P *≤ 0.01 were considered significant with single and multiple testings, respectively.

## Results

### RA patients and controls

Of 160 patients included, 61 (38%) did not complete the four-year protocol. The reasons for drop-out were adverse events (11), treatment failure (10), patients' request (13) and other (27). Fifty-six (35%) left the study during the first two years. Patients who dropped out did not differ from completers with regard to demographic and clinical variables at baseline (data not shown). At baseline one patient had serum SP-D of 8,106 ng/ml. This patient subsequently developed severe pulmonary fibrosis and was excluded from the statistical analyses.

The demographic characteristics of the RA patients at baseline and the control population are shown in Table 1. Among the 142 patients included in the intention-to-treat analyses, all data for composite disease activity measures were available in 134 individuals. Seventy-eight percent, 66% and 69% had achieved ACR50, ACR70 and DAS28 < 2.6 after four years.

**Table 1:** Demographic characteristics of RA patients at baseline and healthy controls

Characteristics	RA patients (N = 160)	Controls (N = 1476)	*P*-value
Gender f/m (%women)	107/53 (67%)	761/715 (52%)	*P *< 0.001
Age in years	53(42 to 63)	38 (29 to 46)	*P *< 0.001
Current smokers (%)	57 (36%)	482 (33%)	*P *= 0.42
Disease duration (months)	3.5 (2.7 to 5.0)	-	-
IgM-rheumatoid factor positive (%)	103 (65%)	-	-
Anti-CCP positive (%)	93 (58%)	-	-
Any SE present (%)	116 (73%)	-	-

Median (inter-quartile range)

Comparison between groups was carried out using Mann-Whitney U-test and Fischers Exact test

Anti-CCP, antibodies against cyclic citrullinated peptides; SE, shared epitopes; RA, rheumatoid arthritis

Including patients with radiographs available at both baseline and after four years (N = 133), 53%, 23% and 49% progressed radiographically according to TSS, JSN and ES score, respectively. Of note, however, radiographic progression at four-year follow-up was small in terms of Sharp/van der Heijde units (median (iqr): TSS 2 (0 to 7) to 5 (0 to 11), JSN 0 (0 to 2) to 0 (0 to 4) and ES 2 (0 to 5) to 3 (0 to 8)).

### Serum SP-D in RA

Baseline SP-D in RA patients was 693 ng/ml (649; 770) vs. 913 ng/ml (879; 945) in controls (*P *< 0.001). This difference persisted after adjustment for age, gender and current smoking status (*P *< 0.001) and was also present at four years after adjustment for confounders (*P *< 0.001). Compared to baseline, SP-D had increased in RA patients at four years (893 ng/ml [810; 1013] vs. 693 ng/ml [649; 770], *P *< 0.001) even when adjusting for gender, age and smoking status (p < 0.001). However, at four years, SP-D was still lower in RA patients as compared to controls with adjustment for confounders (*P *< 0.001). There was no significant correlation between age and SP-D in the RA population (rho = 0.06, *P *= 0.42). Likewise, there was no significant gender difference among RA patients. In contrast, SP-D increased significantly with age in healthy subjects (rho = 0.21, *P *< 0.001), and control males had significantly higher levels of SP-D compared to females (Table 2). Both RA and control smokers had significantly higher SP-D than non-smokers (Table 2). Disease activity markers and HAQ score were inversely correlated to SP-D at baseline (CRP: rho = -0.30, *P *< 0.001, DAS28: rho = -0.23, *P *= 0.003 and HAQ: rho = -0.21, *P *= 0.008). No significant difference in SP-D at baseline was observed between patients with respect to anti-CCP, IgM-RF status or any SE present (*P *= 0.50, *P *= 0.14, and *P *= 0.24, respectively). Furthermore, SP-D did not differ between smoking SE positive vs. non-smoking SE positive patients (*P *= 0.13).

**Table 2:** Baseline surfactant protein D in serum (ng/ml) in smokers and non-smokers and according to gender in patients and controls

	RA-patients	Controls
Men	760 (665;1059)	967 (921;1024)
Women	674 (613;759)	852 (818;902)
*P*-value*	0.09	< 0.001
Smokers	850 (686;1014) (n = 57)	1187 (1099;1293) (n = 482)
Non-smokers	671 (604;738) (n = 101)	827 (802;852) (n = 991)
*P*-value*	0.03	< 0.001

Median [95% CI], *Mann-Whitney U-test

RA, rheumatoid arthritis

The CRP change from baseline to four years (Δ) correlated inversely to the SP-D change (ΔCRP vs. ΔSP-D, rho = -0.39 and *P *< 0.001). We found no association between SP-D and radiographic data including estimated annual progression rate (data not shown). Baseline SP-D did not predict radiographic progression (Total Sharp score) at four years (*P *= 0.46)

### SP-D in synovial fluid and corresponding sera

**Figure 1:**
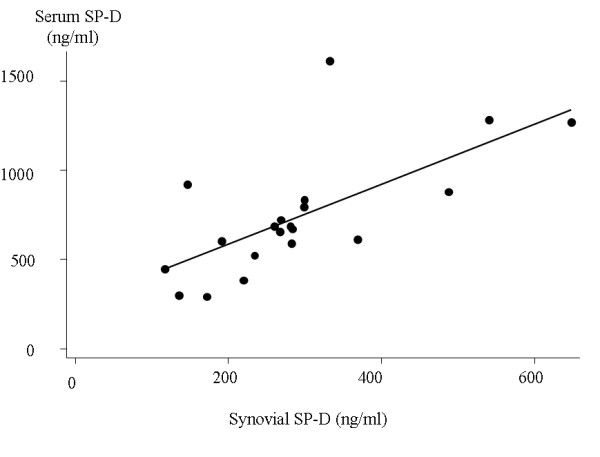
**Scatter plot of SP-D in serum and synovial fluid at baseline (n = 20)**. Fitted values are depicted by the line. SP-D, surfactant protein D.

**Figure 2:**
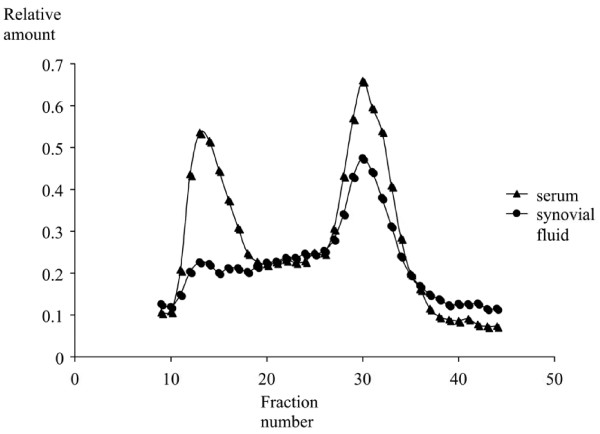
**Size exclusion chromatography of SP-D in serum and synovial fluid**. Mean curves of 11 corresponding serum and synovial fluid samples. SP-D was eluted as two structurally different forms (SP-D multimers (fraction 10 to 18) and SP-D trimers (fraction 24 to 38)). SP-D, surfactant protein D.

Synovial fluid was obtained from 20 patients at baseline. Median SP-D in synovial fluid was 275 ng/ml (221; 299). SP-D in corresponding sera was 678 ng/ml (592; 829). SP-D in synovial fluid and serum levels correlated significantly (rho = 0.69, *P *< 0.001), Figure 1. Synovial fluid SP-D was not significantly associated with sex, age, CRP, autoantibodies, any SE or radiographic findings (data not shown). There was no detectable SP-D in the debris enriched pellets resulting from centrifugation of the synovial fluid. Results from the gel filtration chromatography are outlined in Figure 2. Multimeric SP-D was barely detectable in synovial fluid as compared to serum, where both multimeric and trimeric molecular variant SP-D (trimeric subunits) were detected.

### Genetic SP-D variation in RA

The Met11Thr polymorphism was in Hardy-Weinberg equilibrium in both RA and controls (data not shown). The distribution of genotypes and allele frequencies is presented in Table 4. When adjusting for gender and age, there was no overrepresentation of Thr11Thr in RA patients as compared with controls (Table 3). Circulating SP-D did not differ between genotypes in RA patients, whereas healthy individuals with the Thr11Thr genotype appeared with the lowest level as previously reported [22]. The genotypes were not associated with specific disease features including DAS28, CRP, joint counts, auto-antibodies, HAQ or x-ray findings (data not shown). The Met11Thr allelic variation could neither predict x-ray progression nor disease activity outcome after four years and the size distribution of SP-D in synovial fluid did not differ between genotypes (data not shown).

**Table 3:** Distribution of the SP-D Met11Thr genotype and allele frequencies and corresponding SP-D serum levels (median (95% CI))

	N(%) of RA patients	SP-D ng/ml* RA patients	N(%) of controls	SP-D ng/ml* Controls	*P*-value **	Odds ratio ***
Genotype:						
Met11/Met11	41 (27.3)	724 (636; 1,123)	152 (35.8)	1,081 (996; 1,252)	*P *= 0.16	1.0 (ref)
Thr11/Thr11	27 (18.0)	750 (603; 834)	77 (18.1)	896 (788; 955)		1.3 (0.73; 2.4)
Met11/Thr11	82 (54.7)	660 (563; 761)	196 (46.1)	925 (845;1,023)		1.6 (0.97; 2.6)
						
Allele:						
Met11	164 (54.7)		500 (58.8)		*P *= 0.22	1.0 (ref)
Thr11	136 (45.3)		350 (41.2)			1.2 (0.9;1.6)

* Kruskal-Wallis test: RA patients: *P *= 0.13 and controls: *P *= 0.0023

** Distribution, *P*-value calculated using Fishers' Exact test

*** Odds ratio (95% CI) calculated using logistic regression with health status as the dependent variable and genotype/allele, gender and age as independent variables.

CI, confidence interval; SP-D, surfactant protein -D; RA, rheumatoid arthritis; Ref, reference

## Discussion

Based on the structural similarity between SP-D and MBL and our preliminary report on low circulating SP-D in RA [6], this investigation was conducted to study the possible role of SP-D as disease modifier in RA. While confirming that SP-D in serum is significantly decreased in newly-diagnosed, untreated RA sufferers, we also found an inverse correlation between SP-D and measures of disease activity at baseline. Although SP-D increased significantly during follow-up, it remained subnormal at four years.

The cause of low SP-D in RA is uncertain and different mechanisms may be involved. Altered SP-D expression due to genotype abnormalities should be considered. Thus, in healthy subjects the Thr11-variant is associated with low SP-D in the circulation [22].

In the previous study by Hoegh et al [6], the Thr11 variant tended to be overrepresented in RA patients as compared to controls. This trend was not confirmed in the present study. Thus, a clear genetic contribution to low SP-D in RA cannot be identified in this study. However, a possible genetic contribution to low SP-D in RA cannot be completely disregarded from this study due to the limited sample size. Moreover, it should be borne in mind, that focusing at only one polymorphism in the analysis of gene patterns and serum SP-D, may underestimate the significance of a genetic association, which is better represented by haplotype blocks [35].

Decreased SP-D in RA could be attributable to increased clearance from the circulation, for example, by deposition in inflamed tissues or complex formation with, for example, microbial or cellular waste [36,37]. Thus, cells undergoing apoptosis express auto-antigens, which may lead to auto-antibody formation [38]. Both *in vitro *and *in vivo *experiments have indicated that SP-D enhances clearance of DNA and apoptotic cells by macrophages, thereby reducing anti-dsDNA antibody generation [36,39,40]. Such a scavenger mechanism for SP-D in RA is supported by the inverse association between SP-D and disease activity measures and by the gradual SP-D increase during treatment. The inverse association of SP-D and inflammatory signs and the lack of association between SP-D and erosive progression after four years indicate, that subnormal SP-D is primarily linked to systemic inflammation. According to this, depressed systemic SP-D may contribute to persistent low-grade, subclinical joint inflammation as evidenced by MRI and ultrasonic findings [41,42].

In order to further elucidate the possible role of SP-D in joint inflammation, we quantified SP-D in paired serum and synovial samples and studied the molecular size distribution in serum and synovial fluid. We found a SP-D serum:synovial fluid ratio at approximately 3:1, which indicates that SP-D reaches the joint cavity by diffusion (bulk flow) [43]. The diffusion capacity for proteins across the synovial membrane in rheumatoid arthritis depends on the degree of synovial inflammation and molecular size [43-45]. While both multimeric and trimeric subunit SP-D were present in serum, only trimeric forms could be demonstrated in synovial fluid. This further supports that diffusion is the major source of SP-D in the joint cavity although local degradation of the molecule cannot be excluded. Knowledge about the biologic properties of trimeric SP-D is incomplete. However, previous studies have indicated that trimers interact preferentially with specific microbes, microbial compounds or endogenous lipoproteins [19,46] implying that trimeric SP-D may possess specialized functions as compared with multimeric SP-D. Previously, Gardai et al proposed a model for dual inflammatory activity of SP-D. In the absence of microbial ligands and cell debris, binding of SP-D to macrophages by the CRD region was suggested to be anti-inflammatory by blocking p38 mitogen-activated protein kinases (p38 MAPK) [47]. By contrast, binding of microbial constituents to the CRD region of SP-D would lead to a pro-inflammatory response [47]. Recently, it was shown that posttranslational nitrosylation of cystein residues in the N-terminus of SP-D (SNO-SP-D) caused by inflammation resulted in disruption of multimeric SP-D into nitrosylated trimers. This modified trimeric SP-D variant would subsequently initiate a pro-inflammatory response via calreticulin/CD91 receptor interaction and activate p38 MAPK [48]. Inflammatory signalling resulting in p38 phosphorylation has been identified as an important determinant of synovitis severity [49]. Thus, in theory the dominance of low molecular weight SP-D in synovial fluid observed in the present study may contribute to the maintenance of joint inflammation in RA.

SP-D in serum is suggested to originate primarily from pulmonary leakage [50]. It has previously been demonstrated that smoking increases SP-D in serum [22]. Our findings demonstrate that this also applies to RA patients implying that smoking is a confounder that should be corrected for in the statistical analysis. It has been hypothesized that anti-CCP antibodies can be triggered by smoking through citrullination of lung proteins in SE carriers [51]. We found no correlation between circulating SP-D and SE status in smoking and non-smoking RA patients.

When interpreting the present results, the relatively large number of drop-outs should be considered. However, there was no difference with respect to baseline characteristics between completers and non-completers and the intention to treat analysis included a large majority of the cohort.

SP-D did not correlate to age in RA patients, but tended to be higher in males compared to females. By contrast, SP-D was significantly higher in control males as compared to females, and SP-D correlated positively with age. This disparity may be due to the different sizes of the RA and control populations and the relative overrepresentation of females in the RA cohort. Due to the difference in age distribution in the two populations and rather few controls aged above 50 years we used logistic regression with adjustment for gender and age instead of regular frequency matching in comparisons between controls and patients.

## Conclusions

Circulating SP-D is subnormal at disease onset and after four years treatment in RA. There were no SP-D Met11Thr associations with RA disease activity or subnormal SP-D. While SP-D did not correlate with x-ray progression, we found an inverse association between SP-D and disease activity markers suggesting that low systemic SP-D is involved in the initiation or maintenance of synovitis. Whereas both multimeric and trimeric SP-D variants occurred in serum, only low molecular forms were detected in synovial fluid where it may contribute to joint inflammation. Overall, this study suggests that SP-D is implicated in RA pathogenesis at the protein level.

## Abbreviations

Anti-CCP: antibodies against cyclic citrullinated peptides; CI: confidence interval; CIMESTRA: Ciclosporine, Methotrexate, Steroid in RA; CRP: c-reactive protein; DAS: disease activity score; DMARD: disease modifying anti-rheumatic drug; ES: erosion score; HAQ: health assessment questionnaire; HLA: human leukocyte antigen; IgM-RF: IgM-rheumatoid factor; JSN: Joint Space Narrowing score; MBL: mannan-binding lectin; MTX: methotrexate; RA: rheumatoid arthritis; SE: shared epitopes; SNP: single nucleotide polymorphism; SP-D: surfactant protein -D; TBS: TRIS-buffered saline; TSS: total Sharp-van der Heijde Score; VAS: visual analogue scale.

## Competing interests

The authors declare that they have no competing interests.

## Authors' contributions

All authors contributed to the design of the study, and the acquisition and interpretation of data. AFC performed the statistical analysis. AFC, PJ and GL drafted the manuscript. KJ carried out the immunoassays and gel filtration chromatography. AGJ and AV evaluated the x-ray data. All authors read and approved the final manuscript.
